# Myocardial T2 Star (T2*) in a Large Healthy Population: Correction Factors for a Segmental Approach Using Commercially Available Software in the Current MRI Era

**DOI:** 10.3390/tomography12050075

**Published:** 2026-05-21

**Authors:** Amalia Lupi, Sebastiano Gambato, Ambra Checchetto, Stefania Zinato, Sophie Mavrogeni, Filippo Crimì, Marco Castellaro, Emilio Quaia, Alessia Pepe

**Affiliations:** 1 Institute of Radiology, Department of Medicine—DIMED, Padua University, 35128 Padua, Italy; amalia.lupi@unipd.it (A.L.); emilio.quaia@unipd.it (E.Q.); 2 Department of Information Engineering, Padua University, 35131 Padua, Italy; ambra.checchetto@phd.unipd.it (A.C.); marco.castellaro@unipd.it (M.C.); 3 University Research Institute of Maternal and Child Health and Precision Medicine and UNESCO Chair in Adolescent Health Care, Medical School, National and Kapodistrian University of Athens, Aghia Sophia Children’s Hospital, 10679 Athens, Greece; 4 MRI Unit, Athens Medical Center Palaio. Faliro, 17562 Athens, Greece

**Keywords:** magnetic resonance imaging, iron overload, healthy volunteers, reference values

## Abstract

**Purpose**: Myocardial iron overload has been demonstrated to have a heterogeneous distribution. A segmental T2* CMR approach, with correction factors applied to account for artifacts, has been demonstrated to be feasible and has permitted a reduction in cardiac morbidity and mortality, by better capturing the heterogeneous distribution of myocardial iron overload. To the best of our knowledge, commercially available software does not provide a segmental T2* technique. Our aims were to prospectively examine a large population of healthy volunteers, stratified by sex and age, using the Black Blood MEGE T2* mapping technique, to obtain normative values of the myocardium, to assess their relationship with physiological variables, and to fix correction factors for a segmental approach by using a commercially available software. **Methods**: Fifty healthy subjects (M:F = 1:1, 20–69 years) underwent CMR without a contrast agent. Segmental T2* values were obtained using cvi42 software; global values were the mean. Inter-study, and intra- and inter-operator reproducibility were assessed to confirm the stability of the acquired data. The association of T2* values with physiological characteristics, and myocardial wall thickness were assessed. The fluctuation of all segments versus the mid-septum was calculated to obtain a correction factor for each segment for the software used. Regional T2* differences were examined. A *p*-value <0.05 was considered statistically significant. **Results**: Twenty-five males and females, five for each decade (mean age 43 ± 13.8 years), were included. The native T2* values in all subjects averaged at 34.03 ± 6.65 ms (range 29.9–37.9 ms). Reproducibility analyses showed good correlations between the various datasets (ICC > 0.80). A weakly negative correlation was observed between age and T2* (*p* = 0.04). Segmental correction factors were developed and found to be significantly different from correction factors developed by non-commercially available software on non-state-of-the-art technology for sequences and scanners. **Conclusions**: Age-specific normative values and higher normal cut-off values than the conservative 20 ms are recommended to avoid systematic biases in the identification of pathological findings. Moreover, the correction factors developed by using the most reproducible Black Blood MEGE sequences and a commercially available software on a scanner of the current era could be a significant step toward spreading a more sensitive T2* segmental approach in the clinical arena worldwide.

## Simple Summary

A more sensitive segmental T2* CMR approach has been demonstrated to be feasible, if correction factors for artifacts are applied, and has improved the prognosis in patients with hemochromatosis. Today, commercially available software does not provide a segmental T2* technique. In this study, we evaluated a large population of healthy subjects, and we found a weakly negative correlation between T2* values and age. The segmental correction factors found were significantly different from those developed by non-commercially available software on non-state-of-the-art technology. Age-specific normative values are recommended to avoid systematic biases in the identification of pathological findings. Moreover, the correction factors developed by using state-of-the art sequences and scanners, and a commercially available software, could be a significant step toward spreading a T2* segmental approach in the clinical arena.

## 1. Introduction

T2* Cardiac Magnetic Resonance (CMR) is the gold standard technique for non-invasive myocardial iron quantification [1]. Myocardial iron overload has been demonstrated to have a heterogeneous distribution by autoptic and CMR studies [2]. In a national prospective research network for hemoglobinopathies’ patients (MIOT network), continuous monitoring of cardiac iron and tailored chelation therapy using a segmental approach led to a reduction in cardiac complications and mortality [3]. Nowadays, this more sensitive segmental approach could be particularly valuable also in populations with a known lower iron burden (i.e., primary hemochromatosis, myelodysplastic or politransfused oncologic patients).

For the correct evaluation of the T2* values using a segmental approach, it is necessary to correct T2* values based on signal fluctuations present in healthy subjects due to susceptibility or geometric artifacts [4]. Furthermore, these parameters seem to show a partial dependency on age and sex, along with a possible relation with left ventricular wall thickness.

To the best of our knowledge, to date, T2* assessment by segmental approach is unfortunately not provided by commercially available software and there are no studies that have evaluated the regional and global values of T2* mapping in a large homogeneous cohort of healthy volunteers, using the Black Blood MEGE sequence on 1.5 T equipment in the current era. Black Blood MEGE T2* is the preferred clinical choice for myocardial iron assessment due to its better image quality, reduced artifacts (like blood pool interference), clearer endocardial borders, and improved reproducibility [5].

Thus, the aims of our study were to prospectively examine a large population of healthy volunteers, stratified by sex and age, using the quantitative Black Blood MEGE T2* mapping technique, in order to obtain segmental and global normative values of the myocardium, to assess their relationship with physiological variables, and to fix correction factors for a segmental approach by using a commercially available software.

## 2. Materials and Methods

### 2.1. Study Population

Fifty healthy subjects (M:F = 1:1) were prospectively enrolled between November 2021 and May 2022 and underwent CMR without contrast administration in May 2022, when scanner availability was obtained (2–3 volunteers per day). The inclusion criteria were as follows: age between 20 and 69 years, absence of cardiovascular risk factors, and based on the last biochemical profile available, negative clinical history for either cardiological or systemic disease, no SARS-CoV-2 infection in the 3 months preceding the examination, completion of the SARS-CoV-2 vaccine cycle at least 3 months before the examination, negative electrocardiogram (ECG) in the last week, and absence of absolute contraindications for the execution of an MRI scan. Such criteria were evaluated with an anamnestic questionnaire, and the ECG was performed on-site, if not otherwise available.

The enrollment guaranteed the presence of an equal number of healthy subjects between sexes and for all age groups: 20–29 years, 30–39 years, 40–49 years, 50–59 years, and 60–69 years. If CMR turned out to be pathological, the subject was excluded and replaced with another of the same sex and age group.

All subjects received information regarding the analysis protocol and gave their written consent for the use of their data for clinical and scientific purposes. The study was compliant with the Declaration of Helsinki and was approved by our Institutional Review Board.

### 2.2. CMR Protocol

All examinations were performed on a clinical 1.5 T MRI scanner (MAGNETOM Avanto Fit; Siemens Healthineers, Erlangen, Germany; software version: syngo MR E11), using an appropriate phased-array 18-channel body coil.

Scout images were used to identify the long and short axis of the left ventricle; regarding T2* analysis, three parallel short-axis sections were acquired using Black Blood MEGE sequences, with end-expiration breath holds and cardiac gating in telediastole. The utilized sequence captured images at 10 different echo times: 2.02–22.36 ms with echo spacing = 2.26 ms. Additional parameters were as follows: section thickness = 8 mm; flip angle = 25°; bandwidth = 814 Hz/Px; matrix = 256 × 256 px; and FOV = 400 mm.

A subset of ten subjects, a male and a female from each age group, repeated the CMR protocol to evaluate inter-study reproducibility.

### 2.3. Image Analysis

**Figure 1:**
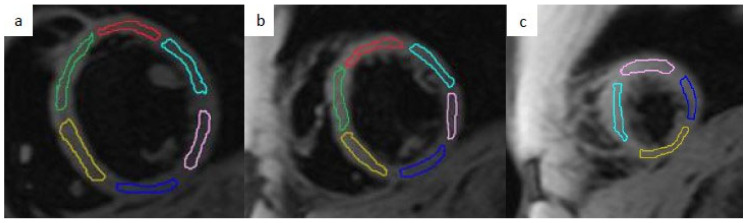
Example of basal (**a**), mid-ventricular (**b**) and apical (**c**) slices of native T2* images examined using cvi42. Manual segmentation of 16 AHA segments by colored ROIs.

All images were transferred to the cvi42 (Circle Cardiovascular Imaging Inc., Calgary, AB, Canada; version 5.11.4) software to determine wall thickness in the mid-ventricular segments, from seven to 12 in accordance with American Heart Association (AHA) segmentation, and to perform T2* mapping analysis. As shown in Figure 1, 16 regions of interest (ROIs) corresponding with the 16 AHA segments were manually traced while applying an offset of up to 2 mm on both the epicardial and endocardial side in order to avoid the epicardial fat and hematic pool for an accurate measure of the segmental T2* values.

**Figure 2:**
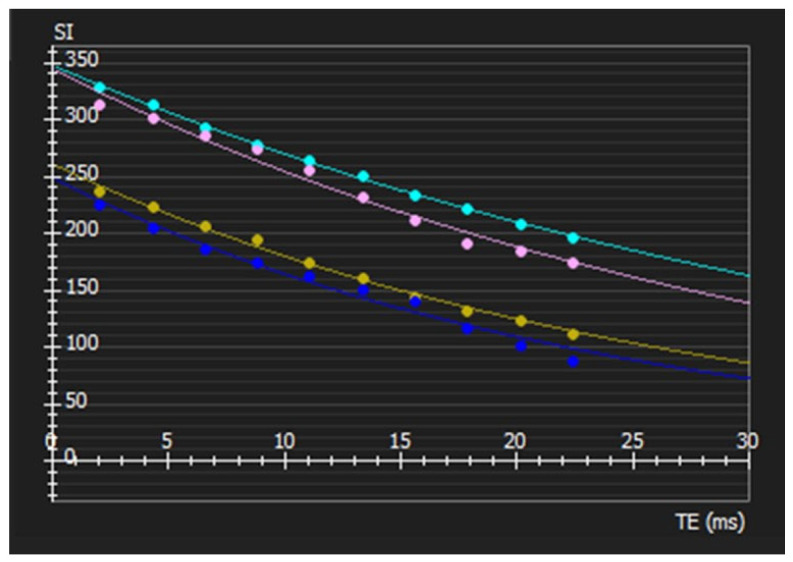
Example of segmental T2* signal analysis of AHA segments 13–16: Segment 13 (pink, T2* = 32.95 ± 1.64 ms), Segment 14 (cyan, T2* = 39.57 ± 0.48 ms), Segment 15 (yellow, T2* = 27.02 ± 0.75 ms), Segment 16 (blue, T2* = 24.24 ± 1.29 ms).

The obtained T2* values were the average of the T2* value of every pixel included within the ROIs, and the global T2* value was the average of all 16 segments’ T2* value; according to the literature and the available software tools, we applied an arithmetic mean (Figure 2).

Image analysis was performed twice by the same observer (S.G., resident radiologist with 1 year of experience in CMR) and again by another observer (A.L., radiologist with 1 year of experience in CMR) on a subpopulation of ten subjects, under the supervision of a cardio-radiologist with more than 20 years of experience in CMR (A.P.), in order to assess intra- and inter-observer reproducibility, respectively.

We assessed the T2* signal fluctuation present in the healthy study population as follows: for each segment, the deviation from the mid-ventricular septum (myocardial region conventionally regarded as protected from artifacts) was evaluated. To calculate a segment-specific correction factor map, T2* values were converted to R2*, and the deviation of the R2* segmental values from the mid-ventricular septum was defined as the difference between the segmental and the mid-ventricular septum R2* value, as previously reported [6], according to the formula

(1) $${∆\langle R2\rangle }_{k}^{*\prime }=\frac{1}{N}\sum_{j=1}^{N}\left(\bar{{R2}_{kj}^{*}}-\bar{{R2}_{msj}^{*}}\right)$$

where the segmental correction factor is defined as the averaged deviations between R2*_kj_ (relaxation rate of the myocardium in a subject j and in a segment *k*) and R2*_msj_ (relaxation rate in the mid-septum R2*).

In this way, a correction factor for each segment was calculated and compared with the previously published correction factors from Positano et al. [6] in the non-commercially available HIPPO MIOT IFC-CNR software.

Moreover, correction factors were validated on an independent cohort of ten subjects without iron overload.

### 2.4. Statistical Analysis

Data were analyzed using R (ver. 4.2.0) and the relevant statistical packages. All continuous variables were defined as mean ± standard deviation (SD), while all categorical variables were expressed as frequencies and percentages.

The normal distribution of the parameters was assessed using the Shapiro–Wilk test for sample size ≤50. The comparison between groups was performed with Student’s unpaired *t*-test or the Mann–Whitney test.

Mean R2* values for each segment were compared with published summary statistics from the HIPPO MIOT IFC-CNR software population [6] through a *t*-test corrected by Bonferroni.

Whenever appropriate, Pearson’s or Spearman’s test was utilized to study the correlation between the T2* values and the physiological variables, specifically age, sex and left ventricular wall thickness. T2* values were analyzed across segments using ANCOVA, with age, sex, and wall thickness as predictors. To mitigate Type I error from multiple comparisons, *p*-values were Bonferroni-corrected.

A two-tailed *p*-value < 0.05 was considered statistically significant.

### 2.5. Reproducibility Analysis

As described below, a subset of ten subjects (one male and one female for each age group) underwent a second CMR exam within a 1 h interval from the first evaluation, and the two sets of T2* images were analyzed to assess inter-study reproducibility. The first set of the same subpopulation images was blindly evaluated a second time by the same operator after at least one week to assess intra-operator reproducibility, and by a second operator to assess inter-operator reproducibility.

After establishing the normal distribution of the acquired datasets, a paired *t*-test or Wilcoxon’s test was applied in order to identify differences in the context of inter-study, intra-operator and inter-operator reproducibility (*p* < 0.05).

The Intra-Class Correlation (ICC) was measured to ensure a good degree of reproducibility; an ICC> 75% suggested an excellent correlation, a 60% < ICC < 74% suggested a good degree of correlation while an ICC < 40% suggested a poor correlation [7].

The inter-study, intra- and inter-operator agreement was evaluated using the Bland–Altman analysis in order to exclude the presence of biases and to calculate the 95% limits of agreement.

## 3. Results

### 3.1. Population

Fifty-two healthy subjects underwent CMR: two subjects were excluded due to pathological findings, such as fatty infiltration/metaplasia (n = 1) and hypertrophic phenotype cardiopathy (n = 1). The 50 subjects composing the study population were of Caucasian ethnicity. Table 1 shows the average age values for the two sexes and the average wall thickness for segments 7–12. Segmental wall thickness ranged from 5.2 mm (mid-ventricular inferolateral segment) to 6.6 mm (mid-ventricular inferoseptal segment); left ventricular wall thickness was statistically higher in male subjects in all evaluated myocardial segments (*p* < 0.001).

### 3.2. Reproducibility Analysis

The mean global native T2* value of all subjects was 34.03 ± 6.65 (range: 20.73–47.33 ms).

Table 2 shows the results of the inter-study, intra- and inter-operator reproducibility of the T2* segmental and global values obtained. The global T2* values showed no statistically significant differences between their mean values (*p* > 0.05), while the variability was significant for the three segmental T2* mean values in the inter-operator analysis.

The ICC for the global T2* was 0.81, 0.94, and 0.89 for inter-study, intra-operator, and inter-operator reproducibility, respectively.

**Figure 3:**
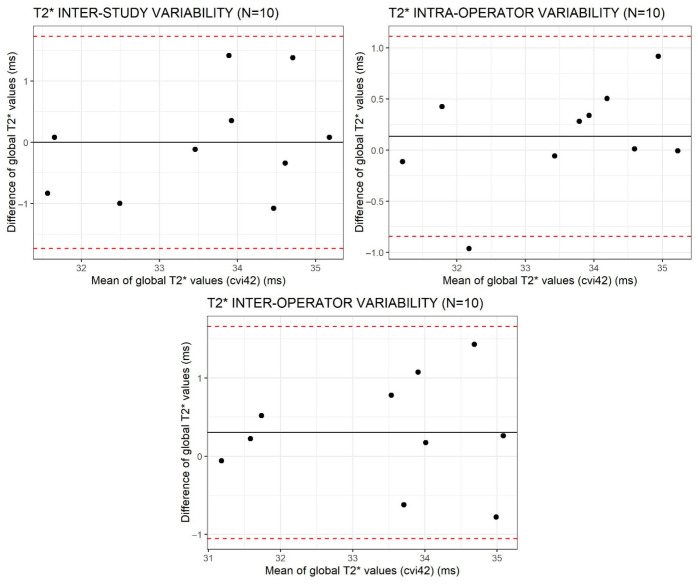
Bland–Altman limits (red dotted lines) for inter-study, intra-operator and inter-operator reproducibility of T2* global values obtained using cvi42.

Bland–Altman plots for the global T2* values are shown in Figure 3.

### 3.3. Correlation Between T2* Values and Population Characteristics

From the correlation analysis between the global T2* values and age, we were able to establish a weak inverse correlation (R = −0.29; *p* = 0.04). The global native T2* values showed no significant differences between male and female subjects (33.8 ± 2.1 ms vs. 34.3 ± 2.8, respectively; *p* = 0.6). Likewise, no correlation between segmental T2* values and relative wall thickness was observed (R = 0.161; *p* = 0.263). The multivariate model implemented did not show a significative combined effect of the variables (*p* > 0.05).

### 3.4. Segmental Variability of T2* Values and Corrections Factors

The analysis of segmental homogeneity showed highly variable T2* ranges, with a difference between two subjects in the same segment that reached 46.1 ms in the inferior distal segment.

**Figure 4:**
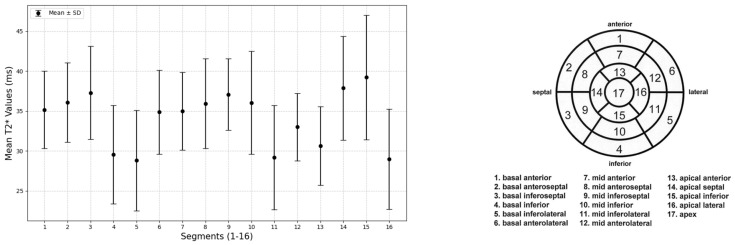
T2* relaxation times (ms) in the 16 AHA segments (**left**); each T2* value is expressed as mean ± SD. Bull’s eye representation of the 16 left ventricle segments according to AHA (**right**).

Using the cvi42 software, which does not correct for the physiological fluctuations of the T2* signal, it was possible to identify segmental differences in the T2* values, shown in Figure 4, with the lowest value recorded in the basal inferolateral segment (28.79 ms) and the highest value in the distal inferior segment (39.2 ms).

The correction factors for each segment are reported in Table 3 and the related R2* values were compared with the known R2* values reported by Positano et al. [6] and used in the non-commercially available software HIPPO MIOT IFC-CNR.

A significant difference was found in eight segments.

Post-correction T2* global values are reported in Table 4.

**Figure 5:**
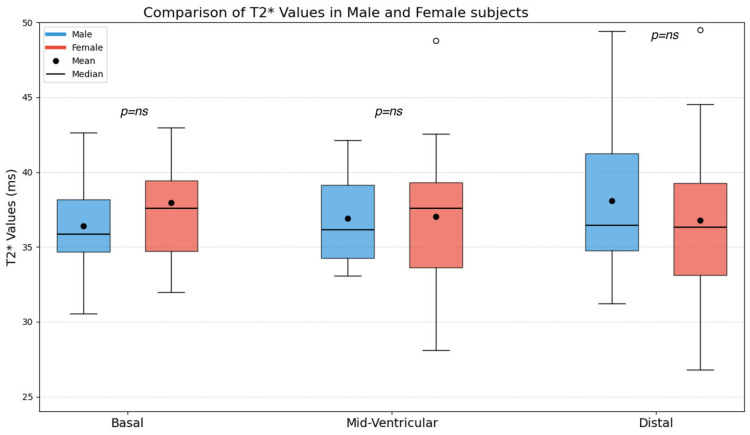
Mean T2* corrected values of basal, mid-ventricular, and distal slices in all subjects, female and male. Ns, not significant. White circles indicate outliers, defined as observations with values exceeding 1.5 times the interquartile range (IQR) above the third quartile.

The mean T2* values obtained in the basal, mid-ventricular, and distal slices were 37.17 ± 3.8 ms, 36.98 ± 3.7 ms, and 37.42 ± 5.0 ms, respectively; no statistically significant differences emerged on basal (36.41 ± 3.2 vs. 37.94 ± 4.2 ms, *p* = 0.16), mid-ventricular (36.92 ± 2.9 vs. 37.04 ± 4.4 ms, *p* = 0.89) and distal (38.08 ± 4.8 vs. 36.76 ± 5.2 ms, *p* = 0.36) slices between males and females (Figure 5).

**Figure 6:**
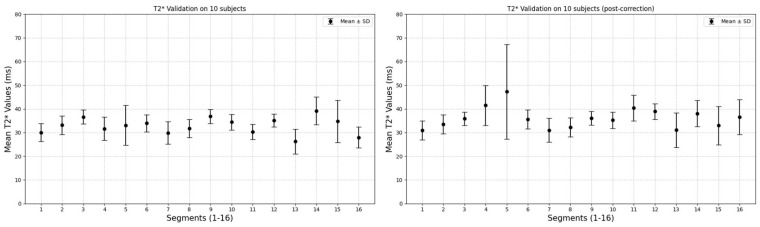
Native (**left**) and corrected (**right**) T2* relaxation times (ms) in the 16 AHA segments in an independent cohort.

Validation results on an independent cohort are reported in Figure 6.

## 4. Discussion

Based on current clinical recommendations [8], a healthy population of 20 subjects is needed to obtain normal mapping reference values, but to the extent of our knowledge, no data are available in the literature regarding the analysis of segmental and global T2* values in such a large and well-stratified cohort of healthy subjects using commercially available software.

A previous study from Positano et al. [6] reported a similar analysis on a smaller healthy population (22 subjects not stratified by sex and age) using a non-commercially available software on white blood MEGE sequences on an old generation 1.5 T GE scanner (GE Signa CV/I; General Electric, Milwaukee, WI, USA). No data are available on the Black Blood MEGE T2* used by Siemens and Philips.

In our study, the reproducibility analysis showed excellent degrees of intra-class correlation of the global T2* values in all cases (ICC > 80% for all datasets), confirming the absence of systematic biases in the repeated measurements performed in different studies and by different operators. The Bland–Altman limits for inter-study reproducibility at segment 11 (mid-ventricular inferolateral) are wide, suggesting poor reproducibility in this region, which is explainable by its more prevalent artifacts. The variability in the inter-individual ranges is likely caused by the sequence construction, which is known to be built to show low variability in the context of pathological values [9]. The results are concordant with previous studies that have well demonstrated the robustness of the T2* technique [10]. The high degree of reproducibility allows us to infer that the T2* values variations can be an expression of specific tissue characteristics, also taking into account the geometric and susceptibility artifacts.

The homogeneity of the segmental T2* values between the three short axis segments appears to reflect the physiological composition of the healthy myocardium.

The effect of the population characteristics on the T2* values was evaluated focusing the attention on age, sex and left ventricular wall thickness. Knowledge of the relationship between these parameters and the T2* values is imperative to ensure a correct application of T2* mapping in the clinical arena. No difference in the T2* values was observed by sex (*p* = 0.6) and no correlation was identified between the T2* values and left ventricular wall thickness (*p* = 0.263); the absence of such correlations is possibly due to the notion that T2* values are mostly dependent on magnetic field variations, which themselves are partly dependent on tissue iron content.

The obtained T2* values showed a weak inverse correlation with age (R = −0.29; *p* = 0.04). Few studies in the available literature have explored this correlation [11], likely due to the small sample size of the studies regarding this topic; in addition, most of them involve younger subjects without a stratified approach based on age. As such, this finding proves to be novel, and possibly dependent on the increase in oxidative stress linked with aging. Thus, cautiously, age-specific normative values can be considered. Nonetheless, these preliminary reference values require validation in a larger cohort, which is needed for the application of age-specific normative values in clinical practice.

Moreover, this study further confirms that the widely used cutoff of 20 ms applied to categorize healthy and pathological findings is inappropriately conservative and carries the risk of misdiagnosing a significant number of borderline patients whose cardiac T2* values range from 20 ms to 27 ms, as is well addressed in post-mortem studies [1,2].

Although no normal reference values are available for BB MEGE sequences, our normal cutoff is comparable to the normative T2* values obtained through a smaller cohort of healthy subjects using white blood MEGE T2* sequences [6,12]. This confirms the known high degree of transferability of the T2* mapping technique, contrary to T1 and T2 mapping, which are known to be scarcely transferable between different scanners [8,9,13].

Through the fluctuation analysis of the T2* signal on our large and healthy study population using the cvi42 software, it was possible to obtain sequence- and software-specific correction factors with respect to the mid-ventricular septum, which represents an adequate indicator of the global T2* value and is conventionally protected from geometric and susceptibility artifacts [1].

The calculated factors constitute the segmental R2* correction map. Application of this map involves subtracting each factor, expressed in R2* (Hz), from the originally estimated segmental R2* value, followed by reconversion to T2* (ms).

Considering the conventional cutoff of 20 ms, the correction map is applied to evaluate whether it results in an increase or a decrease in the segmental T2* values. The greater the absolute value of the factor, the greater the impact of the correction. A positive factor leads to a higher corrected T2*, whereas a negative factor results in a lower corrected value.

From the obtained correction factors, it is possible to observe that the highest values correspond to the basal inferior, basal inferolateral and mid-inferolateral, and apical lateral segments, suggesting that artifacts are more prevalent in those regions, likely due to the pulmonary veins, diaphragm and subdiaphragmatic structures, heart-lung interface and geometric artifacts. In contrast, septal segments of each slice show very low correction factors, consistent with the hypothesis of minimal artifacts in those regions.

To the extent of our knowledge, there is no available literature that describes the correction factors using the Black Blood MEGE and a commercially available software (in the current study cvi42 software).

The R2* values found in our study were significantly different in five segments compared to those used by the HIPPO MIOT IFC-CNR software, in particular in the anterior mid wall, in the whole lateral basal and distal wall, and in the inferolateral mid-wall. The difference is likely attributable to the changed experimental settings. Specifically, the Positano et al. study used:

(a) An older-generation GE MRI scanner with a much smaller gantry diameter, less affected by susceptibility artifacts. Today, the philosophy of all companies is to build larger gantries to reduce the feeling of claustrophobia and to allow even obese patients to be examined.
(b) The use of a MEGE white blood sequence.
(c) A smaller study population, not stratified by sex and age.
(d) A different software.

Thus, the data reported in our study represent a robust experimental model for the development of correction factors in order to implement a T2* segmental approach for myocardial iron quantification in the clinical arena worldwide, and not only in a research setting. The above-mentioned segmental technique will permit sensitive and early diagnosis, extending the prognosis in patients with hemochromatosis [3].

### Limitations

The T2* values gathered in this study were derived from an exclusively Caucasian population; as such, it will be necessary in the future to extend the study to different ethnic groups in order to study potential T2* value differences in various ethnicities.

All the T2* values were derived from an adult population; it is recommended that future studies take into consideration evaluating the T2* values in a pediatric setting.

The study population included a small sample size per age decade, even though, from a clinical point of view, it is the largest population on this issue and in line with current recommendations [8].

The youngest observers had limited experience at the time of the image analyses, although they performed their evaluations under the supervision of an expert observer and after having tested the reproducibility between the youngest observers and the expert observer.

Moreover, CMR acquisitions were carried out by using a single scanner, although the transferability of T2* technique has already been demonstrated. Multicentric studies, preferably using different sequences (white blood MEGE), scanners (Philips and GE), and software should be performed to further confirm our results. Furthermore, external validation of the correction factors in patients with iron overload is recommended. Future studies will not require COVID-specific inclusion criteria, applied in the current study conducted in the historical era of COVID pandemic.

## 5. Conclusions

To the extent of our knowledge, there is no scientific literature regarding the normative global and segmental T2* values in a sex- and age-stratified cohort of healthy subjects. Our data could suggest the implementation of age-specific normative values and higher normal cutoff values with respect to the conservative 20 ms, in order to avoid systematic biases in the identification of pathologic findings, although further confirmations in larger cohorts are recommended. So, we developed correction factors implemented by using the most reproducible Black Blood MEGE sequences and a commercially available software on a scanner of the current era. In this way, we managed to apply a T2* segmental approach in the clinical arena worldwide, and not only in research settings. The above-mentioned segmental technique will permit sensitive and early diagnosis, extending the prognosis of patients with hemochromatosis.

## Figures and Tables

**Table 1:** Characteristics of study population.

	Males (N = 25)	Females (N = 25)	*p* Value
Age	44.1 ± 14.1	42.6 ± 13.9	0.70
Mid-ventricular wall thickness (mm)			
7: mid-ventricular anterior	6.8 ± 1.1	5.0 ± 1.3	**<0.001**
8: mid-ventricular anteroseptal	7.1 ± 1.2	5.3 ± 1.0	**<0.001**
9: mid-ventricular inferoseptal	7.5 ± 1.2	5.7 ± 0.8	**<0.001**
10: mid-ventricular inferior	6.3 ± 1.3	4.7 ± 1.1	**<0.001**
11: mid-ventricular inferolateral	6.0 ± 1.3	4.4 ± 1.0	**<0.001**
12: mid-ventricular anterolateral	5.8 ± 1.0	4.7 ± 0.8	**<0.001**

**Table 2:** Inter-study, intra- and inter-operator agreement of the T2* measurements.

Segment	Inter-Study			Intra-Operator			Inter-Operator		
	Paired *t* Test		Bland-Altman Limits	Paired *t* Test		Bland-Altman Limits	Paired *t* Test		Bland-Altman Limits
	Avg. Difference	*p* Value		Avg. Difference	*p* Value		Avg. Difference	*p* Value	
1: basal anterior	0.29 ± 2.42	0.713	from −4.45 to 5.03	1.14 ± 3.40	0.169	from −5.52 to 7.80	−0.23 ± 1.61	0.662	from −3.38 to 2.92
2: basal antero-septal	−1.13 ± 3.05	0.272	from −7.11 to 4.85	−0.01 ± 1.22	0.980	from −2.39 to 2.37	−0.20 ± 1.36	0.654	from −2.87 to 2.47
3: basal infero-septal	−0.28 ± 2.88	0.766	from −5.93 to 5.37	0.27 ± 1.29	0.525	from −2.26 to 2.80	−0.96 ± 1.52	**0.028**	from −3.95 to 2.03
4: basal inferior	0.13 ± 2.58	0.877	from −4.92 to 5.18	−1.00 ± 2.95	0.312	from −6.78 to 4.78	0.40 ± 2.21	0.580	from −3.92 to 4.72
5: basal infero-lateral	−0.12 ± 2.13	0.862	from −4.29 to 4.05	−0.29 ± 1.61	0.583	from −3.45 to 2.87	0.67 ± 0.75	**0.020**	from −0.79 to 2.13
6: basal antero-lateral	0.70 ± 3.32	0.522	from −5.81 to 7.21	0.77 ± 1.62	0.167	from −2.40 to 3.94	0.75 ± 3.39	0.502	from −5.89 to 7.39
7: mid-ventricular anterior	−0.56 ± 2.99	0.568	from −6.41 to 5.29	0.00 ± 2.19	1.000	from −4.30 to 4.30	0.18 ± 2.35	0.814	from −4.42 to 4.78
8: mid-ventricular antero-septal	1.76 ± 2.75	0.073	from −3.62 to 7.14	−0.34 ± 1.20	0.395	from −2.70 to 2.02	0.38 ± 1.29	0.377	from −2.16 to 2.92
9: mid-ventricular infero-septal	−0.62 ± 2.97	0.525	from −6.43 to 5.19	0.10 ± 0.79	0.699	from −1.45 to 1.65	−0.21 ± 1.72	0.683	from −3.58 to 3.16
10: mid-ventricular inferior	0.57 ± 2.85	0.543	from −5.01 to 6.15	−0.75 ± 1.97	0.260	from −4.62 to 3.12	0.26 ± 2.38	0.737	from −4.40 to 4.92
11: mid-ventricular infero-lateral	0.91 ± 5.58	0.878	from −10.04 to 11.86	−0.81 ± 2.03	0.239	from −4.79 to 3.17	−1.35 ± 2.20	**0.041**	from −5.66 to 2.96
12: mid-ventricular antero-lateral	−0.72 ± 5.20	0.672	from −10.92 to 9.48	−0.17 ± 1.71	0.760	from −3.52 to 3.18	−0.95 ± 2.62	0.281	from −6.09 to 4.19
13: apical anterior	0.42 ± 2.15	0.551	from −3.79 to 4.63	0.34 ± 1.69	0.544	from −2.97 to 3.64	−0.36 ± 1.28	0.396	from −2.86 to 2.14
14: apical septal	−0.39 ± 1.46	0.420	from −3.25 to 2.47	0.19 ± 1.34	0.664	from −2.43 to 2.81	−1.03 ± 2.86	0.284	from −6.63 to 4.57
15: apical inferior	−0.34 ± 2.64	0.693	from −5.51 to 4.83	−0.97 ± 1.46	0.065	from −3.83 to 1.89	−1.85 ± 3.72	0.150	from −9.13 to 5.43
16: apical lateral	−0.57 ± 3.69	0.637	from −7.81 to 6.67	−0.62 ± 2.32	0.420	from −5.17 to 3.93	−0.33 ± 2.99	0.735	from −6.20 to 5.54
global	0.00 ± 0.88	0.991	from −1.73 to 1.73	−0.13 ± 0.50	0.415	from −1.11 to 0.84	−0.30 ± 0.69	0.201	from −1.66 to 1.05

**Table 3:** Correction factors calculated for each AHA segment, and related R2* values compared to those used in HIPPO MIOT software [6]. Ns = not significant.

Segment	Correction Factors Cvi (Hz)	Correction Factors HIPPO (Hz)	R2* Cvi (Hz)	R2* HIPPO (Hz)	*p* Value	Corrected *p* Value
**1. basal anterior**	1.03	4.7	29.0 ± 4.3	32.0 ± 7.9	**0.04**	ns
**2. basal anteroseptal**	0.34	−0.7	28.3 ± 4.5	26.6 ± 5.0	0.16	ns
**3. basal inferoseptal**	−0.54	−1.0	27.4 ± 4.1	26.4 ± 3.8	0.33	ns
**4. basal inferior**	7.29	7.7	35.3 ± 7.2	35.1 ± 8.2	0.92	ns
**5. basal inferolateral**	8.31	3.3	36.3 ± 7.6	30.7 ± 5.9	**0.0031**	**0.0496**
**6. basal anterolateral**	1.36	−2.3	29.3 ± 4.6	25.0 ± 6.6	**0.0022**	**0.0352**
**7. mid anterior**	1.22	6.9	29.2 ± 4.4	34.3 ± 6.5	**0.0002**	**0.0032**
**8. mid anteroseptal**	0.51	0.3	28.5 ± 4.5	27.6 ± 5.4	0.46	ns
**9. mid inferoseptal**	−0.51	−0.3	27.5 ± 4.3	27.1 ± 7.0	0.77	ns
**10. mid inferior**	0.66	4.6	28.6 ± 5.3	32.0 ± 6.3	**0.02**	ns
**11. mid inferolateral**	8.11	0.6	36.1 ± 8.5	28.0 ± 6.3	**0.0002**	**0.0032**
**12. mid anterolateral**	2.82	1.3	30.8 ± 4.0	28.6 ± 3.7	**0.03**	ns
**13. apical anterior**	5.62	6.1	33.6 ± 6.1	33.5 ± 6.7	0.95	ns
**14. apical septal**	−0.78	−2.2	27.2 ± 4.8	25.1 ± 5.1	0.097	ns
**15. apical inferior**	−1.45	−0.2	26.5 ± 5.5	27.1 ± 7.0	0.7	ns
**16. apical lateral**	8.18	2.2	36.2 ± 8.0	29.5 ± 6.6	**0.001**	**0.016**

**Table 4:** Global T2* corrected values for all subjects stratified by age group.

Age Group (Years)	Mean ± SD (ms)
All	37.2 ± 3.1
20–29	38.2 ± 3.6
30–39	36.9 ± 2.7
40–49	38.5 ± 2.7
50–59	37.2 ± 3.2
60–69	35.0 ± 2.5

## Data Availability

The data presented in this study are available on request from the corresponding author (the data are not publicly available due to privacy or ethical restrictions).

## Abbreviations

The following abbreviations are used in this manuscript:

AHA	American Heart Association
BB MEGE	Black blood Multi-echo Gradient Echo
CMR	Cardiovascular Magnetic Resonance
ECG	Electrocardiogram
ICC	Intra-class correlation
MIOT	Myocardial iron overload in Thalassemia
T2*	T2 star
R2*	R2 star
ROI	Region of interest
